# Salivary gland pathologies: evolution in classification and association with unique genetic alterations

**DOI:** 10.1007/s00405-023-08110-w

**Published:** 2023-07-13

**Authors:** Michał Żurek, Łukasz Fus, Kazimierz Niemczyk, Anna Rzepakowska

**Affiliations:** 1 Department of Otorhinolaryngology Head and Neck Surgery, Medical University of Warsaw, 1a Banacha Str, 02-097 Warsaw, Poland; 2 Doctoral School, Medical University of Warsaw, 61 Żwirki I Wigury Str, 02-091 Warsaw, Poland; 3 Department of Pathology, Medical University of Warsaw, 7 Pawińskiego Str, 02-004 Warsaw, Poland

**Keywords:** Salivary gland pathologies, Salivary gland tumours, Salivary gland cancers, Classification, Genetic alterations

## Abstract

**Purpose:**

The correct classification of salivary gland pathologies is crucial for choosing a treatment method and determining the prognosis. Better outcomes are now achievable thanks to the introduction of new therapy approaches, such as targeted therapies for malignant salivary gland tumors. To apply these in clinical routine, a clear classification of the lesions is required.

**Methods:**

The following review examines all changes from the first World Health Organization (WHO) Classification of salivary gland pathologies from 1972 to fifth edition from 2022. Possible developments in the diagnosis and classification of salivary gland pathology are also presented.

**Results:**

The current WHO classification is the fifth edition. With the development of new diagnostic methods, based on genetic alterations, it provides insight into the molecular basis of lesions. This has resulted in the evolution of classification, introduction of new entities and reclassification of existing ones.

**Conclusions:**

Genetic alterations will become increasingly more significant in the identification of salivary gland pathologies in the future. These alterations will be helpful as prognostic and predictive biomarkers, and may also serve as targets for anti-cancer therapies.

## Introduction

Salivary gland pathologies are a range of diverse diseases, therefore, classification is challenging. Moreover, developments in diagnostic methods, particularly at the molecular level, are allowing the discovery of novel subtypes of known diseases, that restrict the proper classification.

The first edition of the WHO Histologic Classification of Salivary Gland Tumours [1] was published in 1972 and included 11 different pathologies divided into three main categories (epithelial tumours, non-epithelial tumours and unclassified tumours). This classification remained in force for almost 20 years until the introduction of the second edition of the WHO Histologic Classification of Salivary Gland Tumours in 1991 [2]. There were 31 pathologies, which were divided into the following categories: carcinomas, adenomas, non-epithelial tumours, malignant lymphomas, secondary tumours and unclassified tumours. Further development of research and improved availability of modern diagnostic methods led to the reclassification of salivary gland diseases in 2005. The third edition of the WHO Classification [3] included 39 pathologies divided into categories: malignant epithelial tumours, benign epithelial tumours, soft-tissue tumours, haematolymphoid tumours and secondary tumours. This classification was in force for 12 years until the fourth edition of the Blue Book was introduced in 2017 [4]. It presented salivary gland lesions in a new perspective, with an emphasis on genetic alterations. Also, new was the proposition of the category 'non-neoplastic epithelial lesions’. In addition, a distinction was made between malignant epithelial tumours, benign epithelial tumours, benign soft tissue tumours and haematolymphoid tumours (a total of 39 pathologies). This was the shortest-lived classification, as only 5 years later, in 2022, the fifth edition of the WHO Classification [5] was introduced. Many key rearrangements in the classification were incorporated. The latest edition also highlights 39 salivary gland pathologies, which are divided into four categories: non-neoplastic epithelial lesions, malignant and benign epithelial tumours and mesenchymal tumours specific to the salivary glands.

The correct classification of a patient’s disease is crucial for choosing a treatment method and determining the prognosis. In the future, the development of modern treatment methods, including targeted therapies in management of malignant salivary glands tumours [6], would provide better treatment outcomes. Worldwide and routine application of such methods in everyday clinical practice will be possible with the accurate and practical classification based on biological and prognostic factors of the lesions for precise identification of patients eligible for a specific therapy.

## Carcinomas/malignant epithelial tumours

**Fig. 1:**
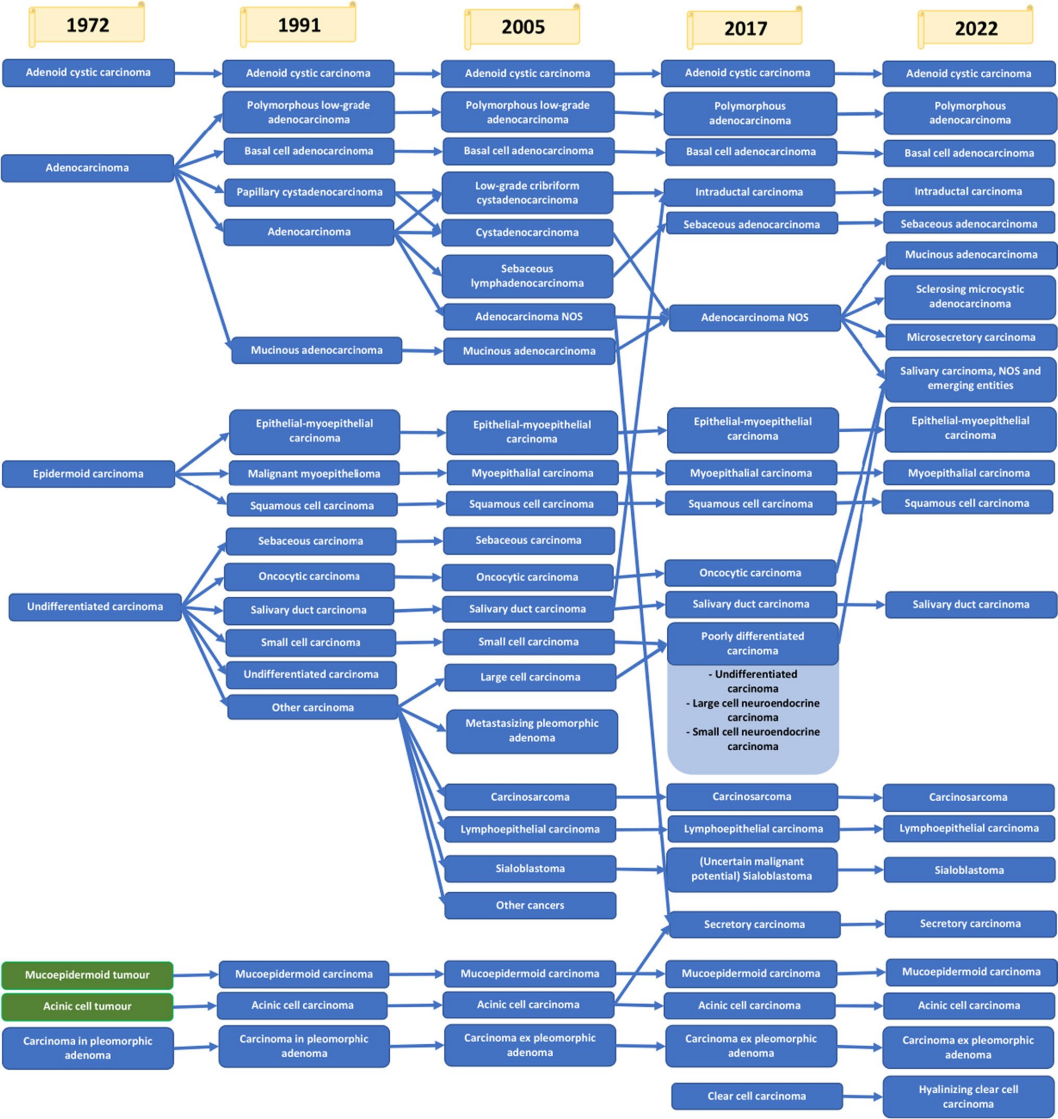
Changes in classifications of salivary gland malignancies

The first edition of the WHO classification of salivary gland diseases distinguished five carcinomas and two tumours (the malignant nature of these was not specified at the time). In subsequent editions, the number of distinguished malignant salivary gland lesions increased. The 1991 classification proposed 18 primary carcinomas. The next edition from 2005 distinguished 24 malignant epithelial tumours. Contrary, the number of malignant epithelial tumours was reduced to 20 types of carcinomas in 2017 and in the following classification from 2022, 21 different malignant salivary gland pathologies were identified. The changes in classifications are shown in Fig. 1.

Some diagnoses have remained the same since 1972. These include adenoid cystic carcinoma and carcinoma in pleomorphic adenoma (or carcinoma ex pleomorphic adenoma). Mucoepidermoid and acinic cell carcinomas were initially classified as tumours of uncertain malignancy, but were recognized as malignant lesions in the second edition of the classification. Although there has not been a reclassification of these lesions over the years, it should be emphasized that the definitions of individual diagnoses have been updated. The first two editions of the Blue Book based the classification on histological features seen with conventional light microscopy. Immunocytochemistry was limited to specific cases [7]. From 2005, immunohistochemical markers started to be introduced into the definitions. In the fourth edition, the importance of translocations and gene fusions was raised. Molecular alterations were included, among others, in definitions of mucoepidermoid and adenoid cystic carcinoma in the latest edition of the Blue Book [5]. The key molecular alterations of salivary gland malignancies are presented in Table 1.

**Table 1:** Selected genetic alterations in salivary gland malignancies [11, 14, 20]

Tumour type	Gene	Mechanism	Prevalence
Acinic cell carcinoma	NR4A3	Fusion/activation	86%
Adenoid cystic carcinoma	MYB	Fusion/activation/amplification	80%
	MYBL1	Fusion/activation/amplification	10%
	NOTCH	Mutation	14%
Basal cell adenocarcinoma	CYLD	Mutation	29%
Carcinoma ex pleomorphic adenoma	PLAG1	Fusion/amplification	73%
	HMGA2	Fusion/amplification	14%
	TP53	Mutation	60%
Epithelial-myoepithelial carcinoma	HRAS	Mutation	78%
Hyalinizing clear cell carcinoma	EWSR1-ATF1	Fusion	93%
Intraductal carcinoma			
Intercalated duct subtype	NCOA4-RET	Fusion	47%
Apocrine subtype	PIK3CA HRAS	Mutation Mutation	High High
Salivary duct carcinoma	HER2	Amplification	31%
	FGFR1	Amplification	10%
	TP53	Mutation	56%
	PIK3CA	Mutation	33%
	HRAS	Mutation	33%
	AR	Copy gain	35%
	PTEN	Loss	38%
	CDKN2A	Loss	10%
Microsecretory adenocarcinoma	MEF2C-SS18	Fusion	> 90%
Mucinous adenocarcinoma	AKT1 E17K	Mutation	100%
	TP53	Mutation	88%
Mucoepidermoid carcinoma	CRTC1-MAML2	Fusion	40–90%
	CRTC3-MAML2	Fusion	6%
	CDKN2A	Deletion	25%
Myoepithelial carcinoma	PLAG1	Fusion	38%
	EWSR1	Rearrangement	13%
Polymorphous adenocarcinoma			
Classic subtype	PRKD1	Mutation	73%
Cribriform subtype	PRKD1	Fusion	38%
	PRKD2	Fusion	14%
	PRKD3	Fusion	19%
Sebaceous adenocarcinoma	MSH2	Loss	10%
Secretory carcinoma	ETV6-NTRK3	Fusion	> 90%
	ETV6-RET	Fusion	2–5%

Essential modifications have occurred in the classification of adenocarcinomas. The first edition of the WHO classification [1] did not distinguish subtypes of this carcinoma at all. In the second edition [2], it was divided into five distinct types. and in the subsequent 2005 edition [3] into 7 adenocarcinoma subtypes. The next classification from 2017 [4] was simplified to four types of adenocarcinomas These changes have given more freedom to pathomorphologists. The grade of the tumours was no longer included in the classification. At the same time, low-grade cribriform cystadenocarcinoma was reclassified into intraductal carcinoma. The latest, fifth WHO classification [5] introduces three new entities—microsecretory adenocarcinoma, sclerosing microcystic adenocarcinoma and mucinous adenocarcinoma.

Despite the development of diagnostic methods and increasingly precise requirements for classifying lesions into a specific type of carcinoma, there are still some difficulties in distinguishing between certain pathologies. Some of these are discussed in the following paragraphs.

An example is the relation between intraductal papillary mucinous neoplasm (IPMN) and mucinous adenocarcinoma. Mucinous adenocarcinoma (regardless of subtype) is characterized by a recurrent AKT1 p.E17K mutation [8, 9]. The same mutation is present in IPMN and the histopathological features resemble mucinous adenocarcinoma [10]. The relationship between the two lesions remains controversial. IPMN can be considered as a separate lesion, precursor or subtype of mucinous adenocarcinoma [5].

Intraductal carcinoma is characterized by proliferations entirely or predominantly intraductal. Some scientific reports state that invasive growth can appear in intraductal carcinoma, so it is not truly in-situ neoplasm and the name “intraductal” may not be correct [11–13].

Oncocytic appearance is common in different salivary gland tumours. Lesions consisting entirely of oncocytes have been classified as oncocytic carcinoma. However, some studies at the molecular level indicate that these lesions should rather be classified as an oncocytic subtype of other carcinomas. To date, neither we have real evidence that purely oncocytic carcinoma exists, nor there have been discovered characteristic genetic alterations for this type of cancer [11, 14–16].

The distinction between primary and secondary squamous cell carcinoma (SCC) of salivary gland still remains a diagnostic challenge. The majority of cases are metastatic tumours [17]. The diagnosis of primary SCC remains a diagnosis of exclusion. The radiological examinations are necessary to identify the site of origin, because it is often not obvious at the time of presentation. It is difficult to differentiate between primary and secondary SCC on histopathology exam [18]. Both are characterized by keratinization. Primary SCCs exhibit a desmoplastic reaction and peritumoral inflammation compared to metastatic SCCs, as well as a serrated margin and less central necrosis [19]. However, these findings are non-specific. Till now, no characteristic biomarkers or genetic alterations specific to primary SCC have been discovered.

## Adenomas/benign epithelial tumours

**Fig. 2:**
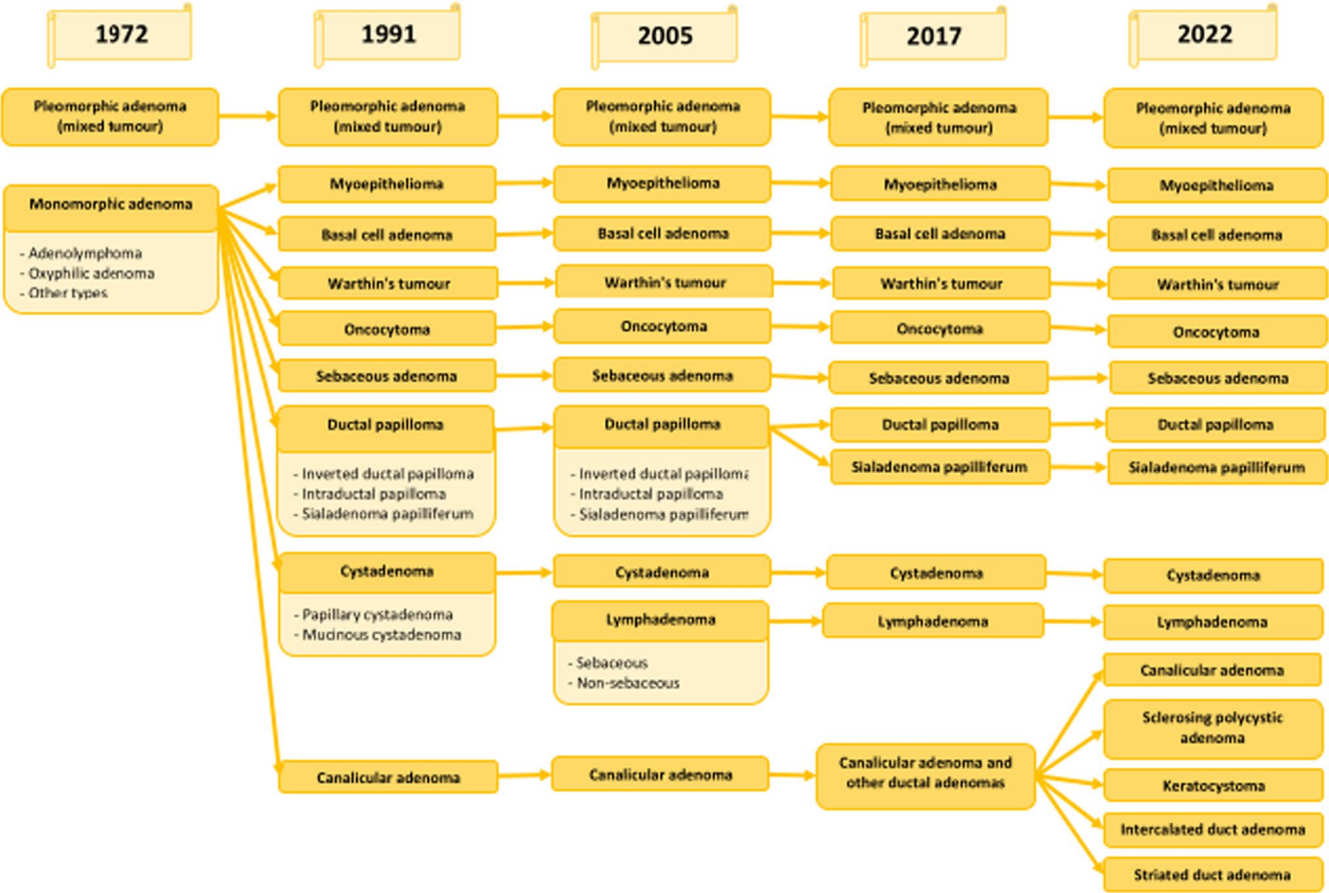
Changes in classifications of salivary benign epithelial tumours

The first edition of the WHO classification of head and neck tumours [1] distinguished two benign salivary gland tumours—pleomorphic and monomorphic adenomas (with subtypes adenolymphoma, oxyphilic adenoma and other types). Subsequent classifications included many more benign lesions—9 in the second [2], 10 in the third [3], 11 in the fourth [4], and 15 in the fifth [5]. The breakthrough between the first and second editions of the Blue Book was due to the increased recognition of benign lesions and distinct morphological features of monomorphic adenomas, so it was decided to separate the lesions for identification purposes (Fig. 2).

Similarly to the malignant lesions, the involvement of genetic differences is also emphasized among benign lesions. Although still none of the benign salivary gland lesions is defined by genetic alterations, specific molecular changes have been identified and may provide an aid to classification and serve as potential biomarkers in the future. The most important genetic alterations in benign salivary gland lesions are shown in Table 2.

**Table 2:** Key genetic alterations in salivary benign tumours [11, 20]

Benign epithelial tumours	Gene	Mechanism	Prevalence
Basal cell adenoma	CTNNB1 AXIN1	Mutation Mutation	37–80% ~ 36%
Myoepithelioma	PLAG1	Fusion	~ 40%
Pleomorphic adenoma	PLAG1 HMGA2	Fusion/amplification Fusion/amplification	> 50% ~ 15%
Sialadenoma papilliferum	BRAF V600E	Mutation	50–100%

Although the classification of benign salivary gland lesions does not pose as many problems as in case of malignancies, the relationship between pleomorphic adenoma and metastasizing pleomorphic adenoma has caused controversy over the last few classifications. Pleomorphic adenoma, also called benign mixed tumour, is found mostly in the parotid gland in third to sixth decade of life, and occurs more frequently in women [21, 22]. It is the most prevalent lesion among salivary gland benign tumours (up to two-thirds of all adenomas) [23, 24], but it is worth noting that recent studies indicate that this lesion is becoming rarer compared to Warthin's tumour, the incidence of which has been increasing recently [25–27], particularly affecting Europe [28]. Pleomorphic adenoma progresses slowly, but can undergo malignant transformation to carcinoma ex-pleomorphic adenoma [29]; rarely can metastasise without the transformation and is called metastasizing pleomorphic adenoma [30]. Metastasizing pleomorphic adenoma is histologically indistinguishable from pleomorphic adenoma [31]. The term was introduced in the third edition of Blue Book as malignant carcinoma, but subsequent classifications have dropped the distinction of this change as a separate entity. The most common genetic alterations in pleomorphic adenomas are PLAG1 and HMGA2 fusions or amplifications [11, 32].

The merit of genetic studies is the manifestation of the neoplastic features of sclerosing polycystic adenoma. This lesion was first introduced in the fourth edition of the WHO classification in the non-neoplastic epithelial lesion category [4]. However, several studies have shown recurrent mutations in the PI3 kinase pathway (primarily PIK3CA mutation), which confirm its neoplastic nature [33–36]. As a result of these findings, the latest classification of salivary gland lesions includes sclerosing polycystic adenoma to benign epithelial tumours [5].

## Others

Other lesions described in the WHO classification included secondary and unclassified tumours, soft tissue tumours, lymphomas and non-neoplastic epithelial lesions.

Non-epithelial tumours were classified since the first edition of the Blue Book [1]. Starting from the third edition in 2005, the name of this group of lesions has been changed to soft tissue tumours and one subtype, haemangioma, has been distinguished [3]. In 2017, lipoma/sialolipoma and nodular fasciitis were added to this category [4]. However, these lesions were omitted from the latest classification [5]. The reason is that they do not occur exclusively or predominantly in salivary glands [11].

Hematolymphoid tumours were firstly added to classification in second edition, and described as malignant lymphomas [2]. There are distinguished lymphomas as part of systemic disease and as separate salivary gland manifestations. The lymphomas were classified using the same terminology as is applied to lymphoid lesions [37]. In the next edition, the name was changed to haematolymphoid tumours and three types of lesions were distinguished [3]. In 2017, this category was restricted to a single diagnosis [4]. The lymphoid tissue is a part of mucosa-associated lymphoid tissue and the extranodal marginal-zone B-cell lymphoma is the most common primary non-Hodgkin's lymphoma of the salivary glands [38]. In the latest edition of the Blue Book, these changes have been deleted from the classification [5].

For the first time in the fourth edition of the Blue Book, a category of non-neoplastic epithelial lesions was introduced [4]. The main diagnosis in this group is sclerosing polycystic adenosis. Lesions of this type had been known since 1996 [39], and the need to add this diagnosis to the classification had already been postulated several years before the fourth edition [40]. Other diagnoses in this category included nodular oncocytic hyperplasia, lymphoepithelial sialadenitis and intercalated duct hyperplasia. In 2022, non-neoplastic epithelial lesions were limited to two diagnoses: nodular oncocytic hyperplasia and lymphoepithelial sialadenitis [5]. Sclerosing polycystic adenosis has been renamed to sclerosing polycystic adenoma and added to the category benign epithelial tumours [11].

**Fig. 3:**
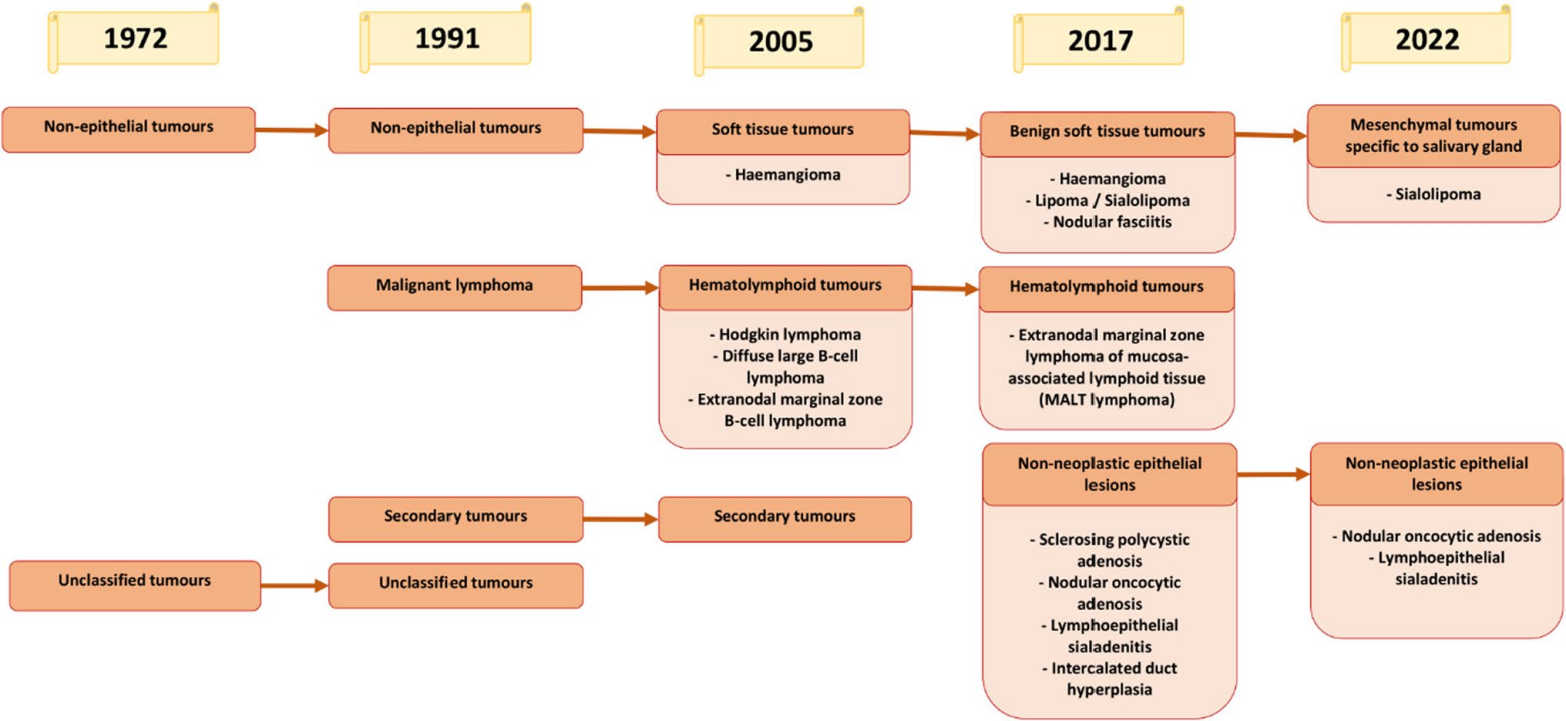
Changes in classifications of other salivary gland entities

A summary of the changes in the classifications described above is shown in Fig. 3.

## Therapeutic and prognostic implications of correct diagnosis

Proper diagnosis of salivary gland pathology allows us to make the right therapeutic decision and determine the patient's prognosis. The most common treatment for salivary gland tumours is surgical resection, and the extent of surgery is determined mainly by anatomical and clinical criteria, but for some lesions an accurate diagnosis should influence therapeutic decisions. In the case of pleomorphic adenoma, the risk of tumour recurrence is about 2–3% and is highest in the myxoid subtype, as well as in the presence of thickness and incompleteness of the tumour capsule, pseudopodia, and satellite nodules [41, 42]. For this reason, more extended surgical techniques are preferred for the treatment of pleomorphic adenoma. Another criterion for extended surgical treatment is recurrence [42, 43].

Accurate differentiation of lesions is also important in planning treatment of canalicular adenomas, which have been divided into five different diagnoses in the latest classification of pathology [5]. Currently lesions classified as canalicular adenomas occur mainly in the upper lip [44], but other lesions are predominantly recognized in the parotid glands. For intercalated duct adenoma, striated duct adenoma and keratocystoma, the prognosis is the best, and no recurrence of the lesions has been described to date [5]. However, in the case of sclerosing polycystic adenoma, there is a risk of recurrence [45] and even malignant transformation of the lesions [40, 46], which should prompt expanded resection technique and more frequent postoperative follow-up. Until 2022, the aforementioned pathologies were not differentiated, which, as indicated above, may be misleading in the treatment and prognosis of patients.

The biggest differences in patient prognosis and treatment standards are seen when comparing different types of malignancies. If there are no contraindications, surgery with total tumour excision is the treatment of choice, according to NCCN Guidelines [47]. Postoperative radiotherapy (RT) should also be considered in all adenoid cystic carcinomas as well as for other malignancies when specific circumstances are found. In most cases of recurrences, RT is also recommended. Recent studies emphasize the role of RT in the management of malignancies of the salivary glands and show improved overall survival in specific subtypes—adenoid cystic carcinoma, adenocarcinoma, high-grade mucoepidermoid carcinoma, and carcinoma ex pleomorphic adenoma [48, 49]. Studies show 5- and 10-year survival rates with different salivary gland malignancies at 52–85% and 32–75%, respectively [49–53]. The best prognosis is for acinic and adenoid cell carcinoma, and the worst for adenocarcinoma and squamous cell carcinoma, with differences in 5-year survival reaching up to 68% [49].

Changes in the latest WHO classification [5] allow a more accurate determination of patient prognosis for less common malignancies. The introduced diagnoses—mucinous adenocarcinoma, sclerosing microcystic adenocarcinoma and microsecretory adenocarcinoma—are mainly located in the intraoral minor salivary glands, and their clinical features include painless mass or swelling [54, 55]. Only in case of mucinous adenocarcinoma recurrence, local and distant metastases are common [54, 56], which should prompt appropriate diagnostic and therapeutic decisions.

The uncovered specific molecular characterization of salivary gland cancers subtypes provides potential for exact definition and diagnosis but also perspectives for development of personalized therapeutic strategies. The described genetic alterations are oftentimes targetable, thus recurrent and metastatic cancers patients are already encouraged to participate in clinical trials. Patients with adenoid cystic carcinoma and MYB overexpression are included in the ongoing MYPHISMO trial with novel vaccination approach, used synergistically with programmed cell death protein 1 (PD-1) inhibitors [57]. In turn, 12 patients with adenoid cystic carcinoma and confirmed activating NOTCH1 mutations were targeted with monoclonal antibody, brontictuzumab, and the phase I study resulted in an objective response rate (ORR) of 17% [58]. The phase II clinical trial ACCURACY evaluated the inhibitor AL101 in patients with recurrent and metastatic adenoid cystic carcinoma and activating NOTCH 1–4 mutations and resulted in the ORR of 15% and disease control rate (DCR) of 65%, determining the inhibitor as promising neoadjuvant setting [59]. Recent studies confirmed detection of prostate-specific membrane antigen (PSMA)-ligand in 93% of adenoid cystic carcinomas, opening perspectives for efficient therapy with ^177^ Lutetium PSMA [60]. In vitro studies with mucoepidermoid cancer models positive for CRTC1-MAML2-positive present sensitivity to EGFR inhibitors, such as erlotinib, gefitinib, or cetuximab, that in the future can be an attractive therapeutic option. The salivary duct carcinoma characterize in high overexpression (78–96%) of androgen receptor (AR) [61] and the treatment has been already supported with androgen-deprivation therapy (ADT; with goserelin). The phase II one-arm study on combined androgen blockade with leuprorelin and bicalutamide in patients with recurrent or metastatic salivary gland cancer proved the ORR of 42% and DCR of 86% with 30.5 months of median overall survival (OS) [62]. Adenocarcinoma is another type with relatively increased load of genetic alterations. AR positive adenocarcinomas were similarly to salivary duct carcinoma targeted with ADT therapy in clinical trials, while HER2 amplified tumours demonstrated enhanced sensitivity to T-DM1 therapy [63].

The rare incidence of other salivary gland cancers subtypes and even the lower rate of metastatic and recurrent cases are so far not conducive to inclusion in clinical trials on systemic therapies.

## Potential developments and trends in salivary gland pathology classification

Initial classifications of salivary gland pathologies focussed on conventional histopathological examination. The second edition of the WHO classification [2] recommended selected immunocytochemical tests—amylase, S-100 protein, actin, myosin, cytokeratin, leukocyte common antigen, carcinoembryonic antigen and thyreoglobulin—for identifying lesions in addition to basic staining. At the time, cytophotometry was an additional test to help differentiate between selected tumour types [7].

A decisive direction in the development of diagnosis and identification of pathologies was introduced in the fourth version of the Blue Book [4], when emphasis was placed on genetic alterations in tumour cells [64]. The new paradigm of genomic alterations is featured heavily for adenoid cystic carcinoma, mucoepidermoid carcinoma, secretory carcinoma, and pleomorphic adenoma [32]. The current edition of the WHO classification introduced commonly reported genetic alterations into the definition of certain cancer types: mucoepidermoid carcinoma, adenoid cystic carcinoma, secretory carcinoma, polymorphous adenocarcinoma, hyalinizing clear cell carcinoma, mucinous adenocarcinoma, and microsecretory adenocarcinoma [5, 11]. The most important genetic variations included in the WHO classification are shown in Tables 1 and 2. Although, the number of salivary gland carcinomas without known molecular alterations has shrunk in last years, there are still a few lesions that remain mysteries. These are basal cell adenocarcinoma, epithelial–myoepithelial carcinoma, sialoblastoma, sclerosing microcystic carcinoma, and sebaceous adenocarcinoma [15]. The reason for these unsolved problems is the rare occurrence of these tumours. However, it is likely that the forthcoming research will soon help to understand the cytopathophysiology of these lesions.

Increasing numbers of researchers are highlighting the importance of genetic alterations as biomarkers of salivary gland pathology. It has been suggested that the genetic changes have also prognostic and predictive potential [14, 65]. Alterations at the genetic level result in changes to the tumour microenvironment. This represents a potential focus for targeted therapies and offers many promising results. Combination of immunotherapies with the antineoplastic agents constitutes a promising approach for the future [66]. Many therapies are still in the early preclinical phase and most of them are described in the review by Mueller et al. [6]. The most potential immunohistochemical biomarkers for underlying molecular changes are presented in Table 3.

**Table 3:** Potential biomarkers in salivary gland tumours [11, 14]

Tumour type	Gene rearranged or mutated	Frequencies	Ancillary IHC
Acinic cell carcinoma	NR4A3	~ 85%	NR4A3
Adenoid cystic carcinoma	MYB	29–86%	Myb
	NOTCH1	~ 14%	NICD
Mucoepidermoid carcinoma	CRTC1	40–90%	Areg
Salivary duct carcinoma	AR	40–70%	AR
	ERBB2 (HER2)	29–35%	Her2
Secretory carcinoma	NTRK (primarily ETV3-NTRK3 fusion)	> 90%	Pan-Trk
Basal cell adenoma	CTNNB1	37–80%	β-Catenin, LEF-1
Pleomorphic adenoma and carcinoma ex pleomorphic adenoma	PLAG1	> 50%	Plag1
	HMGA2	10–20%	Hmga2

Recently, the importance of fine needle aspiration (FNA) cytology in the diagnosis of salivary gland lesions has increased [11]. Although it is a well-known examination that has been used for years [67], only with the introduction of an international standardized FNA assessment system—the Milan system [68]—there has been a return to the widespread use of this test in routine diagnosis of salivary gland lesions. Recently there have been an increasing number of reports of the very high sensitivity and specificity of FNA examination assessed by the Milan system [69, 70]. FNA has the advantage of safety, simplicity of technique and low cost. It is commonly used as an initial diagnostic method. Sometimes, however, non-diagnostic results are reported due to insufficient aspiration or inherent limitations in distinguishing between benign and malignant cytology results [71]. While FNA is cytological, in core-needle biopsy (CNB) a small piece of tissue is taken intact, making it possible to diagnose and stage malignant and benign tumours by examining the histological architecture of the tissue and all its components [72]. In comparison studies, CNB yields significantly fewer non-diagnostic results and has higher sensitivity and specificity than FNA for differentiating malignant and benign salivary gland tumours [71–73]. However, it is known that the risk of complications such as bleeding, pain or tumour seeding is higher for CNB than for FNA [71]. Some authors suggest that the safety profile of CNB conducted by experienced staff and using good-quality equipment is excellent and CNB should be considered the technique of choice when a nodule is detected in the parotid glands [72, 74]. Comparing the development potential of the two methods, it is reasonable to suspect that due to its advantages, FNA will be fostered, but until it achieves comparable sensitivity and specificity results, CNB remains the standard for preoperative testing.

So far none of the WHO classification of salivary gland pathologies includes imaging findings in the diagnosis of the lesions. Radiological examinations are also under constant development, and the utility of new techniques in the diagnosis of salivary gland proliferative lesions has been confirmed in recent studies. The recent significant progress in improving ultrasound imaging with the introduction of new technological solutions as shear wave elastography (SWE) and contrast enhanced ultrasonography (CEUS) influence the preoperative diagnostic workup in salivary gland pathologies. The studies published so far confirm the increased value of SWE versus classic ultrasound in differentiating between the most common benign lesions, polymorphic adenoma and adenolymphoma [75, 76]. Although the amount of studies evaluating the value of CEUS in salivary gland tumours is still low, the presented results are very promising. It has been proven that the mean washout time of the contrast is significantly higher in malignant lesions, while the time to peak enhancement is significantly longer in pleomorphic adenoma than adenolymphoma [77]. Wei et al. [78] proved high combined efficacy of CEUS and Doppler ultrasound in diagnosis of a malignant tumour with the sensitivity of 92.3%, specificity of 86.9% and negative predictive value of 98.5%.

Dynamic contrast-enhanced magnetic resonance imaging (DCE-MRI) could be useful for recognizing the principal types of salivary gland tumours. The study of Mungai et al. [79] affirms DCE-MRI as very valuable biomarker for differentiating benign from malignant tumour. Similarly, Zhang et al. [80] used Haralick texture analysis on computed tomography (CT) imaging of mucoepidermoid carcinomas of salivary glands to determine the tumour phenotype with 89% sensitivity. It is likely that the radiomic biomarkers in the identification of salivary gland lesions will be one of the development pathways for the diagnosis and classification of this type of pathology. The level of advanced capabilities for radiological evaluation and the development of new imaging techniques is a topic too vast for thorough discussion in this article. We point out, however, that the undeniable advantage of imaging examinations is their widespread accessibility in daily clinical work. Perhaps this will become an alternative to expensive genetic testing in the future.

Another perspective for the development of salivary pathology diagnostics is artificial intelligence. The first paper on using machine learning to evaluate salivary gland lesions was published in 2010. Siebers et al. [81] evaluated 10 parameters based on ultrasound of parotid glands of 138 patients differentiating lesions into benign and malignant. They obtained area under receiver operating characteristic curve (AUC) score of 0.91. In the following years, more and more papers addressing this topic were published, and in recent years the topic has become extremely popular, and dozens of original papers and reviews on artificial intelligence in the evaluation of salivary gland tumours are published every year. There is considerable hope for results using machine learning to evaluate ultrasound, CT and MRI images of salivary gland pathology. Wang et al. [82] and then Zhang et al. [83] proved the greater effectiveness of artificial intelligence in distinguishing benign from malignant parotid lesions based on ultrasound compared to experienced clinicians.

Some studies used machine learning to distinguish benign and malignant lesions of the parotid glands based on CT scans [85–87] and MRI images [84, 88, 89] obtaining great effectiveness. Yu et al. [87] developed a deep learning-assisted diagnosis models based on CT images that significantly improved the accuracy of diagnoses of benign and malignant lesions made by experienced radiologists (AUC by 0.128 and sensitivity by 0.194). Chang et al. [90] used deep learning to distinguish Warthin's tumour, pleomorphic adenoma and malignancies of the parotid glands. Not only did the method proposed by the researchers achieve high results (accuracy: 0.71–0.81), but it also detects pathologies on its own and the radiologist does not need to mark the suspicious area on the MRI image, which gives extremely high potential for using the algorithm in clinical practice. Unfortunately, the methodology needs to be improved due to its low sensitivity for detecting malignant lesions (0.33). The number of ongoing research on artificial intelligence models is growing continuously, and the quality of the models is improving. This is a sure direction for the development of diagnostics, and artificial intelligence assisted diagnosis models will certainly become a standard in daily clinical practice in the future.

## Conclusions

Correct diagnosis of salivary gland lesion is essential in determining the treatment and prognosis of the patient. Over the last 50 years, there have been many changes in the classification of salivary gland pathologies and the definitions of several lesions known for years have been updated. The most recent changes concern predominantly genetic studies results, which are increasingly being used in lesion classification. In the future, the importance of genetic alterations in the diagnosis of salivary gland pathology will increase even more. These alterations will also be helpful as prognostic and predictive biomarkers, and may also serve as targets for anti-cancer therapies.

## Abbreviations

WHO: World Health Organisation
IPMN: Intraductal papillary mucinous neoplasm
SCC: Squamous cell carcinoma
MALT: Mucosa-associated lymphoid tissue
FNA: Fine needle aspiration
DCE: Dynamic contrast-enhanced
MRI: Magnetic resonance imaging
CT: Computed tomography
CNB: Core-needle biopsy
ORR: Objective response rate
DCR: Disease control rate
AR: Androgen receptor
ADT: Androgen-deprivation therapy
SWE: Shear wave elastography
CEUS: Contrast enhanced ultrasonography
